# Changes in Biochemical, Strength, Flexibility, and Aerobic Capacity Parameters after a 1700 km Ultraendurance Cycling Race

**DOI:** 10.1155/2014/602620

**Published:** 2014-08-10

**Authors:** Vicente Javier Clemente-Suarez

**Affiliations:** Department of Motricity, Human Performance and Sport Management, Faculty of Sport Sciences, European University of Madrid, Calle Tajo, s/n, Villaviciosa de Odón, 28670 Madrid, Spain

## Abstract

The purpose of the present research was to study the organic response after ultraendurance cycling race. Selected biochemical, leg strength, flexibility, and aerobic capacity parameters were analyzed in 6 subjects 5 days before and 5 days after completing a 1700 km ultraendurance cycling race. After the race, participants presented a significant decrease in Hb (167.8 ± 9.5 versus 141.6 ± 15.7 mg/dL), strength (29.4 ± 2.7 versus 25.5 ± 3.7 cm in a countermovement jump), and oxygen uptake and heart rate at ventilatory threshold (1957.0 ± 458.4 versus 1755.2 ± 281.5 mL/kg/min and 140.0 ± 9.7 versus 130.8 ± 8.3 bpm, resp.). Testosterone presented a decrease tendency (4.2 ± 2.5 versus 3.9 ± 2.6 ng/L) in opposition to the increase tendency of cortisol and ammonium parameters. Transferrin and iron levels presented high values related to an overstimulation of the liver, a normal renal function, a tendency to decrease flexibility, and an increase in aerobic capacity, finding a tendency to increase the absolute maximal oxygen uptake (37.2 ±2.4 versus 38.7 ± 1.8 mL/min) in contrast to previous studies conducted with subjects with similar age. These results can be used to program training interventions, recovery times between probes, and nutritional and/or ergonomic strategies in ultraendurance events.

## 1. Introduction

Previous studies have examined the organic response in endurance running, swimming, triathlon, kayaking and cycling races [1–4]. In recent years, more athletes have become involved in ultraendurance races, such as the ironman triathlon, the 100 km race, and longer races performed in various days [1, 3–5]. Actually, strenuous physical activities are becoming increasingly popular around the world. It is known how ultraendurance events produce an increase in muscle and protein breakdown [4, 5], an increase in the catabolic state of the organism [5], an increase in the erythropoiesis to compensate the exercise-induced haemolysis [6], and an increase in triglycerides consumption [5], have no effect on renal function [1], and are performed with a blood lactate concentration lower than the anaerobic threshold [7].

Moreover in the line of research related to endurance probes, several studies have shown that endurance training produces interference with strength production [8, 9]. Then ultraendurance events could negatively affect strength production capabilities of athlete's muscles. It is also known that an inverse relation between the flexibility and endurance capabilities exists, producing endurance training a decrease on athlete's flexibility manifestations [10]. Related to cardiovascular and endurance performance factors, numerous studies showed that high intensity and low volume training produce higher adaptations in these parameters than low intensity and high volume efforts [11, 12]. Efforts similar than in ultraendurance probes performed in various days.

Therefore, the effect of endurance and ultraendurance events in the athlete's physiological response has been widely studied in one or few days' probes, but the effect of longer ultraendurance events on the athlete's organic response is not known, especially in extreme environmental conditions such as high temperature and humidity. Additionally, to the best of our knowledge, the effect on muscular capacities as strength and flexibility and athlete's cardiovascular function have not been researched in ultraendurance events. For this reason the aim of the present research was to study modifications in selected biochemical, strength, flexibility, and aerobic capacity parameters after completing a 1700 km ultraendurance cycling race. It was hypothesized that an ultraendurance event would alter biochemical markers and would not affect flexibility, strength, and aerobic capacity parameters, since previous studies reported significant changes in biochemical markers after ultraendurance events and secondly only training programs produced changes in flexibility, strength, and aerobic capacity parameters, not only an ultraendurance race.

## 2. Materials and Methods

### 2.1. Experimental Approach to the Problem

**Figure 1:**
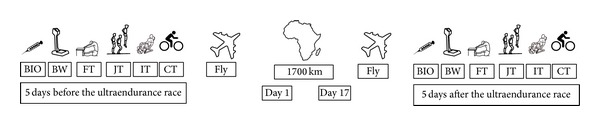
Experimental procedure. BIO: biochemical analysis; BW: body weight analysis; FT: flexibility test; JT: jumps test; IT: isokinetic strength test; CT: cycling test.

A descriptive study was performed. Pre-post changes were analyzed in selected biochemical, strength, flexibility, and aerobic capacity parameters five days before and five days after completing a 1700 km ultraendurance cycling event in order to confirm the study hypothesis.  Figure 1 represents the experimental procedure of the present study. The dependent study variables were body mass; sit and reach value; biochemical: blood urea, creatinine, sodium, potassium chloride, lactate dehydrogenase (LDH), CK, iron, ammonium, testosterone, cortisol, lactate, transferrin, hemoglobin (Hb), triglycerides concentration, and creatinine clearance; strength: peak torque, peak torque/weight, time to peak torque, maximal work, total work, power average at 30°·s^−1^ and 60°·s^−1^ angular velocity in leg extension and flexion movements, squat jump (SJ), countermovement jump (CMJ), and abalakov jump (ABK) performance; aerobic performance: probe time, watts at VO_2max⁡_, VO_2max⁡_ absolute, VO_2max⁡_ relative, maximum heart rate, watts at ventilatory threshold, VO_2_ relative at ventilatory threshold, VO_2_ absolute at ventilatory threshold, and heart rate at ventilatory threshold in a maximal incremental cycling test. The independent variable was the 1700 km ultraendurance event.

### 2.2. Participants

The six participants in the ultraendurance event were analyzed. The athletes were part of an ONG that promoted this sport challenge in the African continent; they were not professional athletes and for this reason the participants were not experienced athletes and parameters like age were higher than in previous studies. The characteristics of the athletes were (mean ± SD) age 56.2 ± 6.9 years; height 170 ± 0.1 cm; body mass 73.3 ± 10.2 kg, body mass index: 25.1 ± 1.5 kg·m^2^, 3.1 ± 1.2 years training cyclist, average of  2.5 ± 1.5 days/week, and 8.2 ± 1.3 hours of training per week. Prior to participation, the experimental procedures were explained to all the participants, who gave their voluntary written informed consent. All were given a medical examination prior to participation to assess their health state and detect any medical condition which might result in injury during the study. The study was conducted in accordance with the Declaration of Helsinki.

### 2.3. The Race

The ultraendurance race consisted of crossing the African continent from the east coast to the west coast by bike. The distance of the race was 1700 km and was performed in 17 days, averaging 100 km per day. The temperature oscillated between 8 and 39°C and the humidity oscillated between 64 and 85%.

### 2.4. Assessment Protocol

In pre and post samples athletes realized the same assessment protocol to measure the study variables. The protocol was as follows.

*Blood Draw in Hospital*. All participants performed blood draw fasting between 9:00 and 10:00 am. Samples for biochemical assay were collected into sterile vacutainer tubes.

*Measurement of Body Mass*. Body mass was analyzed by SECA 222 (Apling, Barcelona, Spain).

*Warm-Up*. A standardized warm-up consists in 10 min of cycling (140 bpm, 90 rpm) in a cicloergometer (Monark 630 Monark Exercise, AB, Sweden) and 2 series of 10 repetitions of submaximal CMJ.

*Flexibility Evaluation*. Sit and reach test [13] was performed 3 times; we analyzed the maximal value reached.

*Leg Strength Evaluation*. Vertical jump test: athletes performed 3 SJ, 3 CMJ, and 3 ABK in an Ergojump System (Bosco System, Ergotest Technology). The rest period between jumps was always 30 seconds. The best jump in terms of height was taken for further analysis. We chose to use vertical jumps as they provide further insight into the force capabilities of leg extensor muscles [14]. Moir et al. [14] suggest that vertical jump assessment in athletes and recreationally active men can be achievedwith a high degree of reliability.

*Isokinetic Leg Strength Evaluation*. The knee extensor and flexor muscle peak torque (absolute) of each leg were concentrically measured at 30°·s^−1^ and 60°·s^−1^ (5 repetitions each) using a Biodex System 3 isokinetic dynamometer (Biodex Corporation, Shirley, NY) according to standard procedures [15].

The athlete was strapped into the chair, using the lateral femoral condyle as an anatomical reference for the axis of rotation. The length of the lever arm was individually determined, and the resistance pad was placed proximal to the medial malleolus. Gravity correction was applied after direct measurements of the mass of the lower limb lever arm system at 30° knee extension. Range of motion varied from 90° knee flexion to 10° extension (considering 0° as full extension). The values of the peak torques over 5 consecutive contractions for each muscle group tested were used for the data analysis. One min of rest was allowed between assessments at different angular velocities using the protocol described by Bradic et al. [16]. All participants indicated that their right leg was dominant. Participants were instructed to hold their arms across the chest to isolate extension movements in knee joint [17].

*Aerobic Capacity Evaluation*. Incremental maximum cycling test was performed in a cicloergometer (Monark 630 Monark Exercise, AB, Sweden) using a CPX gas analyzer (Medical Graphics Corporation, St. Paul, MN). Athletes performed the following incremental test protocol: 5 min 50 w warm-up, increments of 50 w per minute until exhaustion. The incremental test was performed with a cadence between 90 and 105 rpm. To evaluate heart rate a polar S810 (Polar Electro Ibérica. Barcelona, Spain) was used. The test finished when the participant reached at least three of the following five criteria [18]: (a) a plateau in the oxygen consumption (VO_2_) versus exercise intensity relationship, which has been defined as an increase in VO_2_ of less than 2 mL/kg/min with an increase in exercise intensity, (b) elevated respiratory exchange ratio (*r* ≥ 1.0), (c) elevated HR (≥90% of [220-age]), (d) a rating of perceived exhaustion (RPE) of 19-20 on the Borg scale, and (e) high levels of blood lactate concentration (≥8 mmol/L).

### 2.5. Study Variables

The following parameters were evaluated in pre and post samples.

Biochemical parameters: blood (25 mL) was withdrawn from an antecubital vein, using a sterile technique to analyze parameter of urea (mg/dL), creatinine (mg/dL), sodium (mmol/L), potassium (mmol/L), chloride (mmol/L), LDH (UL/L), CK (UI/L), iron (*μ*g/dL), ammonium (*μ*mol/L), testosterone (ng/L), cortisol (*μ*g/dL), and transferrin (mg/dL). The analyses were performed on an Olympus AU 800 Autoanalyzer. Results were corrected for changes in plasma volume as previous research [19]. 32 *μ*L capillary bloods from fingertips were collected to analyze blood lactate concentration (mmol/L), Hb (mg/dL), and triglycerides (mg/dL). Blood lactate was measured using an Accusport Lactate Analyzer (Total Performance Inc., Mansfield, Ohio). This portable lactate analyzer has been found to be valid and reliable [20]. Hb and triglycerides were analyzed by a Reflotron Plus system (Roche Diagnostics S.L., Sant Cugat del Vallès, Barcelona, Spain). Creatinine clearance (mL/min) was estimated by Cockroft and Gault formula that has shown a good correlation with glomerular filtering [21].

- Body mass (Kg).
- Vertical jump parameters: jump height in SJ (cm), CMJ (cm), and ABK (cm).
- Isokinetic parameters: peak torque (n·m), peak torque/weight (%), time to peak torque (mseg), maximal work (J), total work (J), and power average (w) in knee extensor and flexor muscle of the legs at velocities of 30°/seg and 60°/seg.
- Flexibility parameter: sit and reach performance (cm).
- Aerobic capacity parameters: probe time (seg), watts (w) at maximal oxygen uptake (VO_2max⁡_), VO_2max⁡_ absolute (mL/min), VO_2max⁡_ relative (mL/kg/min), maximum heart rate (bpm), watts at ventilatory threshold (w), VO_2_ absolute at ventilatory threshold (mL/min), VO_2_ relative at ventilatory threshold (mL/kg/min), and heart rate at ventilatory threshold (bpm).

### 2.6. Statistical Analysis

The SPSS statistical package (version 17.0; SPSS, Inc., Chicago, Ill.) was used to analyze the data. The Shapiro-Wilk normality test was used to test the normality and homogeneity of each variable. All data presented a nonparametric distribution; therefore a Wilcoxon *t*-test was performed to compare prerace and postrace data. In order to improve the applicability of the research to exercise professionals the effect size (ES) was tested by Cohen's *d* test and interpreted according to Rhea classification for recreationally trained athletes [22]. This classification was proposed for determining the magnitude of training interventions that commonly produced a small range of change due to the exquisiteness of training programs required to elicit adaptations [23], especially in small sample sizes or large variance data [22] as in the present study. The level of significance for all the comparisons was *P* < 0.05.

## 3. Results

Modifications of biochemical parameters analyzed are shown in Table 1. The increase of transferrin and triglycerides presented a large ES as well as the decrease of Hb. However, the increase of iron and ammonium and the decrease of ammonium and creatinine clearance presented a moderate ES. Only the decrease in hemoglobin was significant (*P*: 0.043).

Body mass before the race was 73.3 ± 10.2 kg and after was 71.6 ± 8.5 kg (−1.78%; *Z*: −1.753; *P*: 0.080; ES: −0.016). Flexibility values did not significantly decrease after the race (Pre: 2.8 ± 6.3 cm versus Post: 1.0 ± 2.9 cm; *Z*: −0.674; *P*: 0.500; ES: −0.28). Regarding jump tests only CMJ values significantly decrease (−13.5%) with a moderate ES (Table 2).

None of the values of isokinetic strength conducted at 30°/seg presented significant differences. Isokinetic strength values are shown in Tables 3 and 4.

By contrast, total work and power average in flexion at 60°/seg significantly decreased (−23.2%; *P*: 0.043 and −45.0%; *P*: 0.043, resp.). Time to peak torque in flexion presented a large ES and peak torque/weight, power average and total work, a moderate ES.

The variables of probe time and watts at VO_2max⁡_ in the incremental cycling test significantly increased (8.5%; *P*: 0.043 and 11.4%; *P*: 0.025, resp.), and VO_2_ and HR at VT decreased significantly (−10.3; *P*: 0.043 and 6.6%; *P*: 0.043, resp.) (Table 5). Additionally, probe time and watts in VO_2max⁡_ presented a large ES and HR at ventilatory threshold, a moderate ES.

## 4. Discussion

The purpose of the present study was to analyze modifications in selected biochemical, strength, flexibility, and aerobic capacity parameters after a 1700 km ultraendurance cycling race. A significant decrease in Hb, strength, VO_2_, and HR at ventilatory threshold was measured. The hypothesis of the study was partially supported because when aerobic capacity improved, strength parameters decreased and flexibility and the majority of the biochemical parameters were not modified.

### 4.1. Biochemical

The electrolytes concentration (sodium, potassium, and chloride) measured postrace was similar to the basal sample, despite the event being performed in high condition of humidity and temperature. These findings could be related to the fact that electrolyte replacement strategies were correct and could replace the losses suffered during the race, result similar to previous studies in ultramarathon runners [24]. Results obtained in the sodium concentration rule out any possible hyponatremia, and the unmodified serum potassium values obtained after the race were in consonance with previous researches conducted in marathon [25], 56 km run [26], and triathlon [27]. Regarding renal function indicators such as urea, creatinine, and creatinine clearance, they were not significantly modified. The lack of change of creatinine values coincides with the results obtained after running a marathon, a 100 km running race or a 110 km cycling race [28], but is opposite to an increase measured after a 60 km mountain bike race [1], a 460 km cycle race [5], a marathon [29], a 100 km run [30], and a 24 hour event [31]. This increase in creatinine has been related to the reduced renal blood flow, reduced glomerular filtration rate, and hypovolemia produced in these shorter events [32] and could be related to the higher intensity of these races compared to the 1700 km cycling race. Therefore, the renal function of athletes in the current study was not affected despite the 17 days of race duration.

The variables related to muscle breakdown (CK, LDH, and urea) presented after 5 days values close to the ones obtained in the prerace sample. These data showed that in five days athletes muscles have time to recover despite the high levels of muscle breakdown that usually are measured in these ultraendurance events [4]. It could be due to the fact that the race was performed cycling, which is an activity with no impact and produces a minor damage in the muscle structure. Another parameter traditionally used to control the organic anabolic-catabolic balance, the blood testosterone, presented decreased tendency after the ultraendurance race. This is because the organism still recovering to the catabolic situation that supposed the ultraendurance race, fact also corroborated by the increased tendency in cortisol values [33]. Then, after 5 days the cortisol and testosterone concentrations did not return completely to the basal values; this catabolic status also was reflected in the increased tendency of ammonium values; parameter increased after exercise and related to training workload and effort performed by athletes [34]. In addition, testosterone values measured both before and after the ultraendurance event were lower than in other studies conducted in ultraendurance events, a fact that could be explained because these athletes were younger than in the present research and their testosterone production was higher because of their lower age [35].

The increase in transferrin and iron (large and moderate ES, resp.) after the ultraendurance race could be interpreted as a symptom of haemolytic anaemia that is related to the decrease in Hb [6]. Also the increase in these values could be explained because of the liver overstimulation [27, 28, 30] that increases the production of hepatic enzymes that cause an increase in iron levels. In addition, related to substrates metabolism, triglycerides presented an increase with a large ES after the ultraendurance race that could be due to the discharge of catecholamines induced by the exercise, which stimulated lipolysis in the adipose tissue and led to a release of lipid substrates including triglycerides [4]. Finally, the drop in the Hb concentration was in contrast to the results obtained after an alpine marathon [1] and also after a 20 hour and 51 min cycling event [5], showing that, in the ultraendurance race analyzed, erythropoiesis was insufficient to compensate the breakdown of red blood cells caused by the extreme effort [6] and possibly related to the extreme ambient condition (temperature and humidity) of the race [36].

### 4.2. Strength

The decrease of strength parameters after the ultraendurance race might be due to athletes losing muscular mass because of the stress of the continuous effort [36], a fact that could be related to the body mass loss after the race. Another cause of the decrease in strength parameters might be due to the interferences between endurance exercise and strength manifestation [8]. In this line, the decrease in isokinetic leg strength values was similar to the study of Glowacki et al. [9] after performing a low intensity aerobic training. Also, the study of Abernethy [37] found a decrease in isokinetic leg strength and tension after acute endurance activity in athletes. This fact could be explained because oxidative-endurance training causes muscle to respond in an opposite fashion by ultimately degrading and sloughing myofibrillar protein to optimize oxygen uptake kinetics as shown by Kraemer et al. [8] in a group of subjects to develop endurance exercise.

### 4.3. Flexibility

Participants presented a decrease tendency in flexibility values after the ultraendurance race. This might be due to the constant repetition of a cyclic movement for long time periods. This repetitive movement might cause degeneration in muscle cells that prevented them from showing the initial length. The decrease in flexibility values was in consonance with the results obtained in endurance runner, who presented a decrease in flexibility values because of musculotendinous structures reducing the aerobic demand of submaximal running by facilitating a greater elastic energy return during the shortening phase of the stretch shortening cycle [10].

### 4.4. Aerobic Capacity

A general improvement in aerobic capacity was measured. Athletes decreased HR and VO_2_ at ventilatory threshold, which reflects an improvement on aerobic energy system since participants consumed less oxygen at ventilatory threshold; therefore the energy demand at this intensity was lower [38]. An increase in the HR efficiency was also observed, because athletes performed the same intensity, intensity corresponding to the ventilatory threshold, with a lower HR [39]. These improvements at the intensity of the ventilatory threshold were similar to that obtained after training programs with periods between 12 weeks [40] and 20 weeks [41].

It has been documented that a progressive decline in VO_2max⁡_ with age seems to be due to both central and peripheral adaptations, primarily reductions in maximal heart rate and lean body mass [42]. However, athletes in the current research have managed to present an increase tendency in absolute VO_2max⁡_ values; therefore even in this age, this parameter can be increased in opposition to previous literature [36].

It is also noteworthy that athletes achieved increases in aerobic capacity despite the low intensity effort performed during the ultraendurance race. Previous literature postulated that high intensity training may reduce the decrease in VO_2max⁡_ related to age [43], but the results obtained in the present research demonstrated that long extended aerobic exercise also could improve VO_2max⁡_ of athletes with ages in which previous research only measured decreases. The increase in VO_2max⁡_ might be explained because of the increase in the maximum heart rate [44] and also could affect the initial performance level of athletes that was not high since they only trained 2.5 ± 1.5 days per week.

### 4.5. Limitation of the Study

The principal limitation of the study was the low number of participants analyzed, which limits the generalization of the results obtained in the present research. It was because only 6 participants completed the 1700 ultraendurance race, which is a low number of subjects analyzed, but it represents the 100% of finisher athletes of the race. Also the test conducted after the ultraendurance race could be realized immediately after the race to analyze the acute organic response and also repeated 7 and/or 10 days after the race to analyze the evolution of the different variables analyzed. The testing procedure was conducted in a laboratory in Spain and we had not the option to conduct the tests in the African continent. Then, participants had to fly from Africa to Spain and for this reason we did not conduct the tests immediately after the race, and for lack of funds we cannot repeat the tests in posterior days.

### 4.6. Practical Application

The results obtained in the present study have demonstrated the effect of an ultraendurance event in different organic parameters. These data can be used to program different training interventions, such as the inclusion of supplementary strength sessions to prevent a decrease in muscle strength when high volume and low intensity aerobic effort are performed; additionally, the recovery times between ultraendurance probes and nutritional and/or ergonomic strategies can be implemented to prevent, for example, participant's weight loss, which could lead to their overtraining states.

It has also been shown that low intensity high volume aerobic efforts produce improvements in aerobic performance markers, factor to consider since currently research in this area has shown the effectiveness of high intensity and low volume training. Possibly the concentration of high volumes may also produce improvements in aerobic fitness as well as high intensity and low volume efforts.

## 5. Conclusion

Participants analyzed in the present study presented after five days of completing a 1700 ultraendurance race a significant decrease in Hb, strength, VO_2_, and HR at ventilatory threshold. Testosterone presented a decrease tendency in opposition to the increase tendency of cortisol and ammonium parameters. Transferrin and iron level presented high values related to overstimulation of the liver, a normal renal function, a tendency to decrease flexibility, and an increase in aerobic capacity, finding a tendency to increase the absolute VO_2max⁡_ in contrast to previous studies conducted with subjects with similar age.

## Figures and Tables

**Table 1:** Pre and post mean and SD values of the studied biochemical variables.

Parameter	Unit	Samples		% change	*Z*	*P*	Cohen' *D*
		Prerace	Postrace				
Urea	mg/dL	32.3 ± 7.3	29.5 ± 2.0	−8.6	−.674	.500	−0.38
Creatinine	mg/dL	1.1 ± 0.2	1.1 ± 0.2	0	−.674	.500	0.00
Sodium	mmol/L	144.0 ± 0.8	143.3 ± 2.1	−0.5	−.816	.414	−0.87
Potassium	mmol/L	4.5 ± 0.2	4.5 ± 0.1	−1.1	−.447	.655	0.00
Chloride	mmol/L	106.3 ± 1.0	106.5 ± 1.3	0.2	−.272	.785	0.20
LDH	UI/L	424.8 ± 36.3	411.8 ± 47.3	−3.1	−.730	.465	−0.36
CK	UI/L	81.0 ± 23.3	82.3 ± 32.8	1.5	−.674	.500	0.06
Iron	*μ*g/dL	132.0 ± 27.4	172.8 ± 60.6	30.9	−1.461	.144	1.49
Ammonium	*μ*mol/L	62.4 ± 17.1	78.0 ± 13.3	25.0	−1.095	.273	0.91
Testosterone	ng/L	4.2 ± 2.5	3.9 ± 2.6	−6.5	.000	1.000	−0.12
Cortisol	*μ*g/dL	14.3 ± 2.3	14.6 ± 0.6	2.5	−.674	.500	0.13
Transferrin	mg/dL	188.8 ± 7.3	204.5 ± 17.1	8.3	−.674	.500	2.15
Hemoglobin	mg/dL	167.8 ± 9.5	141.6 ± 15.7	−15.6∗	−2.023	.043	−2.76
Triglycerides	mg/dL	86.2 ± 9.1	127.0 ± 32.2	70.5	−1.753	.080	4.51
Creatinine clearance	mL/min	143.3 ± 17.3	128.0 ± 28.8	−10.7	−.674	.500	−0.87
Lactate	mmol/L	2.3 ± 0.6	2.2 ± 0.3	−3.5	.000	1.000	−0.17

*P < 0.05 versus prerace sample. Ck: creatinine kinase; LDH: lactate dehydrogenase.

**Table 2:** Mean and SD values of the jump test variables.

Parameter	Unit	Samples		% change	*Z*	*P*	Cohen' *D*
		Prerace	Postrace				
SJ	cm	23.3 ± 4.5	22.7 ± 3.4	−2.5	−.405	.686	−0.13
CMJ	cm	29.4 ± 2.7	25.5 ± 3.7	−13.5∗	−2.023	.043	−1.44
ABK	cm	34.6 ± 6.5	29.6 ± 5.0	−14.4	−1.753	.080	−0.77

*P < 0.05 versus prerace sample. SJ: squat jump; CMJ: countermovement jump; ABK: abalakov jump.

**Table 3:** Pre and post isokinetic legs strength at 30°/seg velocity data.

	Parameter	Unit	Prerace	Postrace	% change	*Z*	*P*	Cohen' *D*
Extension	Peak torque	n.m	170.0 ± 27.0	162.4 ± 18.8	−4.5	−.674	.500	−0.28
	Peak torque/weight	%	235.7 ± 37.5	224.4 ± 14.1	−4.8	−.674	.500	−0.30
	Time to peak torque	mseg	674.0 ± 25.1	636.0 ± 61.1	−5.6	−1.355	.176	−1.51
	Maximal work	J	124.6 ± 26.8	124.0 ± 17.6	−0.4	−.135	.893	−0.02
	Total work	J	550.5 ± 123.2	529.3 ± 70.4	−3.9	−.135	.893	−0.17
	Power average	w	43.5 ± 7.2	46.2 ± 5.8	6.3	−.405	.686	0.38

Flexion	Peak torque	n.m	89.6 ± 25.0	87.8 ± 16.5	−2.0	−.405	.686	−0.07
	Peak torque/weight	%	123.4 ± 34.1	120.6 ± 16.0	−2.3∗	−.135	.893	−0.08
	Time to peak torque	mseg	886.0 ± 252.8	1090.0 ± 339.7	23.0∗	−.674	.500	0.81
	Maximal work	J	81.5 ± 28.3	80.7 ± 20.6	−1.0∗	−.135	.893	−0.03
	Total work	J	354.3 ± 129.4	331.9 ± 130.8	−6.3∗	−.135	.893	−0.17
	Power average	w	27.1 ± 10.5	22.7 ± 11.5	−17.9∗	−.405	.686	−0.42

*P < 0.05 versus prerace sample.

**Table 4:** Pre and post isokinetic legs strength at 60°/seg velocity data.

	Parameter	Unit	Prerace	Postrace	% change	*Z*	*P*	Cohen' *D*
Extension	Peak torque	n.m	153.2 ± 38.3	141.4 ± 34.4	−7.7	−1.214	.225	−0.31
	Peak torque/weight	%	209.5 ± 35.2	194.8 ± 46.2	−7.0	−1.214	.225	−0.42
	Time to peak torque	mseg	434.0 ± 84.4	444.0 ± 27.0	2.3	−.135	.892	0.12
	Maximal work	J	118.8 ± 33.5	115.9 ± 26.3	−2.5	−.135	.893	−0.09
	Total work	J	514.7 ± 165.6	451.0 ± 138.4	−12.4	−.405	.686	−0.38
	Power average	w	73.4 ± 22.5	77.4 ± 23.2	5.4	−.405	.686	0.18

Flexion	Peak torque	n.m	85.2 ± 16.5	96.9 ± 32.2	13.8	−.405	.686	0.71
	Peak torque/weight	%	117.1 ± 14.1	133.0 ± 41.0	13.6	−.405	.686	1.13
	Time to peak torque	mseg	476.0 ± 71.3	938.0 ± 480.3	97.0	−1.753	.080	6.48
	Maximal work	J	78.8 ± 16.8	87.9 ± 34.2	11.5	−.674	.500	0.54
	Total work	J	318.8 ± 88.6	244.9 ± 129.3	−23.2∗	−2.023	.043	−0.83
	Power average	w	44.8 ± 13.6	24.6 ± 14.3	−45.0∗	−2.023	.043	−1.49

*P < 0.05 versus prerace sample.

**Table 5:** Mean and SD values of the aerobic performance variables analyzed.

Parameter	Unit	Samples		% change	*Z*	*P*	Cohen' *D*
		Prerace	Postrace				
Probe time	seg	705.4 ± 23.6	765.2 ± 23.2	8.5∗	−2.023	.043	2.53
Watts at VO_2_ max	w	220.0 ± 10.0	245.0 ± 10.0	11.4∗	−2.236	.025	2.50
VO_2_ max absolute	mL/min	37.2 ± 2.4	38.7 ± 1.8	3.9	−1.473	.141	0.63
VO_2_ max relative	mL/kg/min	2706.4 ± 296.9	2749.0 ± 209.1	1.6	−.674	.500	0.14
Maximum heart rate	bpm	173.2 ± 5.7	176.6 ± 7.1	2.0	−1.841	.066	0.60
Watts at VT	w	140.0 ± 20.0	135.0 ± 12.2	−3.6	−1.00	.317	−0.25
VO_2_ absolute at VT	mL/min	26.6 ± 4.3	24.5 ± 1.7	−8.0	−1.214	.225	−0.49
VO_2_ relative at VT	mL/kg/min	1957.0 ± 458.4	1755.2 ± 281.5	−10.3∗	−2.023	.043	−0.44
Heart rate at VT	Bpm	140.0 ± 9.7	130.8 ± 8.3	−6.6∗	−2.023	.043	−0.95

*P < 0.05 versus prerace sample. VO_2_: oxygen uptake; VT: ventilatory threshold.
